# The Cellular Theory

**Published:** 1868-09

**Authors:** A. W. Stein


### ARTICLE X.

### *The Cellular Theory.*

Read before the Medical Journal Association, June 5th, 1868.

By Dr. A. W. Stein.

Dr. Stein began by referring to the immense strides made by medical science during the last half century, through the establishment of the cell theory, which has created the department of histology, given a new foundation for physiology, revolutionized pathology, and thrown a flood of light on therapeutics.

Although the study of minute anatomy has been carried on for several hundred years, the confusion which characterized early microscopic investigations, owing to the imperfect state of optical instruments, prevented the results from being of any great value to the profession. To Schleiden and Schwann is due the credit of having laid the foundation in 1837-8 upon which has been erected the cell theory of 1868. They were the first to establish the doctrine that all organized beings are developed from cells. The data furnished by Schleiden in 1837 with reference to the mode of development in vegetable cells, led to the correction of many erronous views entertained at that time upon this subject. Although previous to this, the resemblance between vegetable and animal tissue had vaguely been referred to, it was left for Schwann in 1838 to demonstrate in a systematic manner, the complete accordance between the cells of animal tissue and those of plants.

A cell is a body, varying greatly in shape and size, though usually of a round or oval form, consisting of a delicate transparent membrane, the cell wall, and enclosing certain contents of a transparent finely granular appearance, called the nucleus or cytoblast; and, in the midst of this latter, a granular body, larger than the rest, known as nucleolus, though this in many young cells is not recognizable. Of the various theories of cell development which have been proposed, those of Schwann, Virchow and Beale, deserve special prominence. Schwann, announced in 1838 the theory of spontaneous generation, or the development of cells out of a formative fluid or blastema (cytoblastema of Schleiden.) He held that out of an amorphous granular fluid (blastema) a granule, the nucleolus, is percipitated, upon the surface of which a stratum of minute granules is deposited, and thus the nucleus is formed: by the accretion around this of new matter, which in due time becomes condensed into a membrane, a complete cell is produced. Virchow in 1858, entirely refuted Schwann's blastema theory, and substituted the doctrine: "Omnis cellula e cellula;" his fundamental axiom being that "The cell is the ultimate morphological element in which there is any manifestation of life, and that we must not transfer the seat of real action to any point beyond the cell." He classifies tissues in three groups: 1st, cellular tissue, such as epidermis, membranes, etc., consisting exclusively of cells in immediate juxtaposition; 2d, connective tissue, comprising the areolar, adipose, white fibrous, yellow elastic, fibro-cartilage, cartilage, bone and dentine, and consisting of cells separated by inter-cellular substances derived from the cell contents, of varying chemical constitution, but having the common character of yielding gelatine when boiled; 3d, compound tissue, comprising the muscular and nervous fibres and blood vessels. The phenommena of contractility, sensibility, and secretion, in muscles, nervs and glands, are essential attributes of cell contents. That the nucleus is the centre of nutrition is evinced by the fact that cells which lose their nuclei, such as blood corpuscles or the superficial layer of cells of the epidermis, have a transitory existence. That it is, so to speak, the organ of reproduction, is shown by the manner in which cells multiply. The first indication of the transformation of the cell is that the nucleolus (if present) becomes constricted in the middle and divides into two nucleoli. This is followed by a like constriction and segmentation of the nucleus, and finally the cell itself becomes constricted between the separated nuclei, and ultimately divides into two cells, appropriating half of the cell contents to each nucleus. A less frequent form of development, peculiar to cartilage, is the formation of secondary cells within existing cells (endogenous cell formation.) Cartilage cells are described as having, like vegetable cells, two membranes; a relatively thick external capsule, analogous to the cellulose covering of plants, and an internal delicate membrane, the primordial utricle. In this instance multiplication is effected in a manner similar to that just described. Virchow affirms that every kind of new formation, pathological as well as physiological, has its origin in the proliferation of the cell; that the inherent activity of the cell is to a great extent independent of nervous vascular influence. He opposes the doctrine that the origin of organic activity is in the nervous system, and adduces the fact that vegetable physiology is based upon the investigation of the activity of cells. The activity of cells is influenced by local excitation or irritation, denominated, according as it may excite one or the other action, functional, nutritive, and formative irritability.

That functional activity is independent of nervous influence is shown by the action of woorara, which destroys the irritability of nerves without affecting that of the muscles; by the contractility, mentioned by Virchow, of many small vessels which, although abounding in muscular fibres, are destitute of nerves; and by experiments upon glands, in which, after section of all the nerves, increased secretion was provoked by injecting irritating substances into the blood. Nutritive Irritability, which is the power possessed by individual parts of taking up and transforming a certain quantity of matter under definite stimuli, is a purely physiological act when just sufficient to mantain the existence of the part; but when matter is taken up in excess, it becomes the seat of special formative changes, and belongs to the type denominated Formative Irratability. That this is independent of nervous or vascular influence, but rather due to special action of the cells, is assumed from phenomena observed in parts destitute of both nerves and vessels. A thread drawn through a cartilage will cause enlargement, through increased absorption of material, in all the cells which lie close to it, while more remote cells remain altogether unaffected. These facts render untenable the theory that the swelling and tumefaction of inflammation is due to increased afflux of blood to the part and an exudation therefrom. Virchow describes two forms of inflammation: the purely parenchymatous, (non-exudative,) and the secretory, (exudative.) In the parenchymatous form no free exudation ever exists, the cells themselves by increased absorption of the transuded fluid of the blood becoming larger, fuller, and filled with a quantity of matter. Secretory (or exudative) inflammation is peculiar to the superficial organs (serous and mucous membranes;) but even here there is at no time a direct fibrinous exudation from the blood. Fibrin, like mucus, is a local product of the tissue in which it is found, and is simply brought to the surface by the transudation of fluid from the bloodvessels. Virchow reduces the elements of pathology heterologous and homologous formations, and holds that into pathological growths no structure of an absolutely new form obtains, but if examined at seasonable time, neither too soon nor too late, there will be found for it a physiological prototype in some parts of the economy. The distinction between homologous and heterologous formatious is that while the former, although new formed, still reproduce the type of its parent cell, as in fibrous tumor arising in a hypertrophied uterus; the latter, although its histological elements may also correspond to some definite normal tissue, is heterologous in deviating from the type of the part in which it arises, e. g. cartilaginous exostosis (enchondroma.)

Dr. Lionel Beale ignores the cell as a true cell, and divides organic matter into germinal matter and formed material, the formor being the living, active, formative element, the latter dead, passive, and formed of the germinal matter. Germinal matter, from whatever source obtained, is composed of a mass of compound particles of a granular appearance, among which may be discovered a nucleus and nucleolus, apparently of the same structure as that in which they lie. The apparent discrepancy between the statements of Virchow and Beale may be reconciled by studying the function of the several constituents of the cell and its development, as illustrated by the following table:

| *Virchow.* | *Beale.* |
| --- | --- |
| Nucleolus,<br>Nucleus,<br>Cell contents, | Germinal matter, |
| Cell walls,<br>Inter-cellular substances. | Formed material. |

According to Beale, germinal matter is the seat of growth.

nutrition, and multiplication in all elementary parts, the nucleus having the power of appropriating crude nutritive material and converting it into living matter like itself. Inasmuch as the nutritive material must reach the most central part (or nucleolus,) it never exceeds one one-thousandth of an inch in diameter without sudividing into separate centres of growth. While the pabulum is constantly passing from the circumference to the centre, the elaborated germinal matter passes in the same ratio from the centre to the circumference, fulfilling in its transit the successive functions of nucleolus, nucleus, and cell contents. On reaching the circumference it becomes fixed and passive, forming Virchow's cell walls and intercellular substances. This process is said to be followed in glands during the function of secretion, the oldest particles of germ matter of each mass being transformed into the substances upon which depend the peculiar properties of a tissue, or the secretion of a gland. M. Ollier has shown that if the germinal matter of one tissue be transplanted into another, it will not produce the tissue in which it is placed, but that from which it was taken, The relative proportion of germinal matter to formed material varies in the different elementary structures and especially at different stages of their development, being greater in young tissues and diminishing with age. As the epithelial cell of epidermis approaches the surface, the nucleus or germinal matter gradually disappears, and cell finally becomes a mere scale. This doctrine of "life from life" is now almost universally endorsed. Professor J. Hughes Bennet, however, still adheres to the molecular theory of organization, holding that all tissues are formed by the successive production of histogenetic, or formative, and histolytic, or disintegrative, molecules: "First molecular depositions in organic fluids; these aggregate to form nuclei, cells, and other tissues; and these again, by constantly breaking down and re-forming, gradually elaborate and build up the higher organisms."

Dr. Stein concluded by urging the importance of histological investigaitons, and expressing his regret that the practical duties of the profession should absorb our energies at the expense of scientific researchcs in America.—*Med. Gazette.*

